# The impact of a digital wheeze detector on parental disease management of pre-school children suffering from wheezing—a pilot study

**DOI:** 10.1186/s40814-021-00917-w

**Published:** 2021-10-09

**Authors:** Stephanie Dramburg, Ellen Dellbrügger, Wim van Aalderen, Paolo Maria Matricardi

**Affiliations:** 1 Department of Pediatric Respiratory Medicine, Immunology and Critical Care Medicine, Charité - Universitätsmedizin Berlin, Augustenburger Platz 1, 13353 Berlin, Germany; 2 Pediatric Pulmonologist, Berlin, Germany; 3 Department of Pediatric Respiratory Medicine and Allergy, Emma Children’s Hospital, Amsterdam UMC, University of Amsterdam, Amsterdam, The Netherlands

**Keywords:** Pre-school wheezing, Digital health, Childhood, Asthma self-management, Wheeze detector

## Abstract

**Background:**

Viral airway infections are a major reason for doctor’s visits at pre-school age, especially when associated with wheezing. While proper treatment requires adequate recognition of airway obstruction, caretakers are often struggling with this judgment, consequently leading to insufficient or late treatment and an unnecessary discomfort of the patient. Digital technologies may serve to support parental decision taking. The aim of the present pilot study is to acquire data on the feasibility of recruitment and observation procedures for a randomized controlled trial on the impact of a digital wheeze detector in a home management setting of pre-school wheezing.

**Methods:**

This single-armed pilot study enrolled patients with a doctor’s diagnosis of wheezing aged 9 to 72 months. Participants were asked to use a digital wheeze detector (WheezeScan, Omron Healthcare, Japan) 2×/day for 30 days and record the child’s respiratory symptoms, detection of wheezing, and medication intake via an electronic diary (eDiary) app. Demographic and clinical data were collected at the recruitment visit. The asthma control test and the Parent Asthma Management Self-Efficacy Scale (PAMSES) were assessed both, at recruitment and follow-up.

**Results:**

Twenty families were recruited and completed the monitoring. All but one completed the follow-up after 30 days. The recruitment procedures were feasible, and adherence to daily monitoring reached an average of 81%. The use of the wheeze detector was rated as uncomplicated. Parents detected wheezing without digital support in only 22/708 (3.1%) of the recorded events. By contrast, the wheeze detector indicated an airway obstruction in 140/708 (19.8%) of the recordings.

**Conclusion:**

In parallel to feasible recruitment procedures, we observed good usability of the wheeze detection device and high adherence to eDiary recording. The positive outcomes show that the WheezeScan may empower parents by increasing their capacity for wheeze detection. This deserves to be investigated in a larger randomized controlled trial.

## Key messages

- What uncertainties existed regarding the feasibility? The feasibility of a digital support device for wheeze detection in a home care setting is currently unknown. Furthermore, the adherence of caretakers to continuous symptom and medication monitoring over several weeks was uncertain before performing the present pilot study.
- What are the key feasibility findings? Twenty families were recruited to this pilot study, and 19 of them completed all study visits and monitoring period (95% retention baseline to post-intervention). Adherence to symptom and medication recording was variable, but caretakers did record at least once per day for 81% of the days. They further reported a high sensitivity but overall good usability of the wheeze detector.
- What are the implications of the feasibility findings for the design of the main study? The intervention was well-received by most participating families. Technical support and reminders for reporting are required to ensure a succesful monitoring period. Future trials should recruit in more than one clinical center in order to increase the diversity of the study cohort.

## Introduction

Viral infections of the upper and lower airways, as well as wheezing, are the first causes of doctor’s consultation in the first 3 years of life [1]. Their social and economic burden at worldwide level is enormous [2, 3]. Young families, especially in Western societies and concerning their first-born child, are not trained to face the regular consequences of upper and lower respiratory tract infections (URI and LRI) as well asthma exacerbations. Therefore, especially infections of the respiratory tract are usually over-treated with unnecessary use of antibiotics accompanied often by an exceeding number of doctor consultations, workdays lost, and decreased quality of life due to stress [4, 5].

Prompt and proper treatment of wheezing is considered beneficial to reduce the burden of lower respiratory diseases. In addition to a good compliance to anti-inflammatory medication, if prescribed, proper recognition of respiratory symptoms, including wheezing, is essential for the consequent adequate decision whether to administer reliever medication. Empowering and educating parents in the self-management, treatment, and control of their child’s wheezing disorder is an important target pursued by pediatricians [6]. However, this capacity is not easily acquired by parents and both over and undertreatment with reliever medication has been observed [7, 8].

Several digital technologies have been or are currently being developed to support parents and health professionals, especially at the primary care level, in the management of wheezing disorders. These include symptom diaries [9, 10] and asthma action plans accessible within mobile health applications for smartphones [11], adherence support via gamification [12], digital therapeutics such as smarthalers [13], or digitally connected diagnostic tools like wirelessly connected peak flow meters [14], digital stethoscopes for health care professionals [15] or mobile wheeze [16, 17], and cough detectors [18]. The market for mobile phone applications is growing remarkably every year, but no quality control system is in place to distinguish guideline-based medical support from arbitrarily compiled or simply wrong content [19]. In contrast, smart devices for digital diagnostics and therapeutics need to be registered as medical devices with a thorough risk assessment. Unlike the registration process for drugs, there is no need to prove efficacy or superiority over existing technologies for medical devices. Observational studies in a real-life setting are essential not only to test whether digital medical devices truly improve disease management or quality of life, but also to enable health care professionals giving adequate recommendations to patients and their caretakers

Recently, a digital wheeze detector (OMRON WheezeScan) has been registered as a medical device in Europe based on a good performance and safety profile as well as solid accuracy in measurements compared to the results of specialized physicians [17]. The aim of the present pilot study was to assess the following aspects of the study design and/or protocol:

- Feasibility of the study protocol and recruitment procedures: the pilot study aimed at assessing whether the use of a digital wheeze detector in a home care setting of pre-school wheezing is feasible and well-accepted by participating families.
- Usability of the WheezeScan device: the study design also included an evaluation of the usability of the device and whether any technical obstacles occurred.
- Usability of symptom and medication monitoring via a mobile application: the adherence to and acceptance of digital symptom and medication recording via a study app were analyzed.

## Materials and methods

### Participants and setting

To address the abovementioned objectives, 20 children aged 9–72 months with a doctor’s diagnosis of wheezing during the last 12 months, requiring a  prescription of reliever medication were recruited in the private practice of a Berlin-based specialist in pediatric pulmonology. The study protocol was approved by the local ethics committee (reference number: EA2_069_20).

### Inclusion and exclusion criteria

Children and their caretakers were recruited according to the following inclusion criteria: (1) at least one episode of doctor’s diagnosed wheezing and/or recurrent cough requiring treatment with beta-2-agonists in the last 12 months, (2) age between 9 and 72 months, (3) sufficient comprehension of the German language, (4) availability of a smartphone (Android or iOS), and (5) consensus to participation. A participation was not possible in one or more of the following exclusion criteria applied: (1) an anatomic malformation causing chronic nasal and/or bronchial obstruction, (2) a severe chronic disease, (3) a contraindication for the use of beta sympathomimetic drugs, and (4) an intention to move away from Berlin during the monitoring period.

### Recruitment

Potential participants were identified by the study doctor during the routine clinical visits. If a child was eligible for the pilot study according to the inclusion and exclusion criteria, the study doctor informed the family about the study and handed out a written information sheet. At the earliest 24 h later, parents with an interest in participation were invited to a recruitment visit. During this visit, all remaining questions were answered, and the parents signed the informed consent form.

### Intervention

After the recruitment visit (T0), all families were asked to use the digital wheeze detector WheezeScan (OMRON Healthcare Co., Ltd.) twice per day (in the morning and evening) and at the same time points to monitor their child’s respiratory symptoms in a mobile clinical diary on their smartphone for a total of 30 days.

#### Digital wheeze detector

**Fig. 1:**
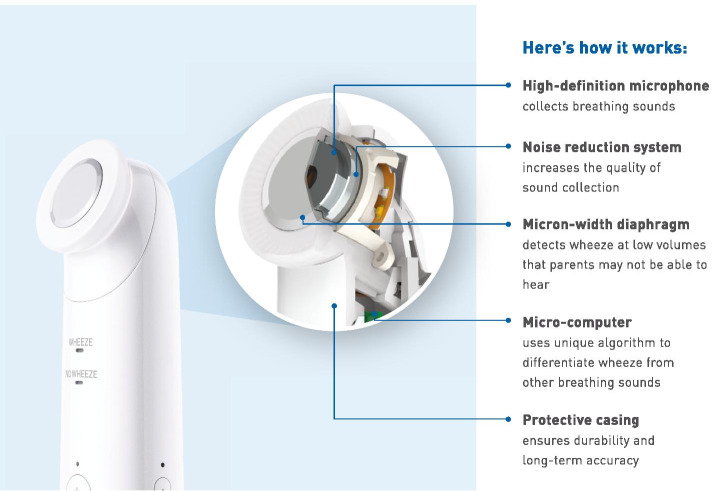
The WheezeScan wheezing monitor device for home use

To detect the presence or absence of wheezing in a home care setting, we used the CE-certified WheezeScan device by OMRON Healthcare Co., Ltd. (Fig. 1). The digital wheeze detector device is developed and tested [17, 20] to record and analyze lung sounds in children and to indicate the presence or absence of wheezing. The measured result is indicated on an integrated display that can be transmitted with a timestamp to a PC or mobile device (e.g., smartphone or tablet computer) via Bluetooth. Further, the WheezeScan stores the date, time, and measurement results in the memory of the device, even if the results are not shown on the device itself. For privacy reasons, no sounds or ambient noise are being recorded. In case of disturbance by ambient noise, the device indicates an error and the measurement should be repeated.

#### Digital symptom diary

The mobile application WheezeMonitor (TPS Software Production S.r.l, Rome, Italy) was used to record respiratory symptoms regularly, at least twice per day. In the morning (7–10 am), in the evening (6–10 pm), and in the case of an exacerbation (any time), parents were asked to fill a short questionnaire on respiratory symptoms (wheezing, Ronchi, cough, shortness of breath) of their child, their decision on the application of reliever medication (beta-2-agonists), the WheezeScan result, and the behavior of the child during the measurements. In addition, the evening questionnaire also contained questions on the retrospective evaluation of the past 24 h regarding symptoms, potential unscheduled doctor’s visits, missed days at school/daycare, and the intake of medication. Reminders automatically appeared on the parent’s phone if a routine questionnaire (morning/evening) had not been filled. If questionnaires were not filled for more than 3 days in a row, parents were contacted by the study team via phone to ask whether the parents needed assistance or technical problems had occurred.

### Measures

**Fig. 2:**
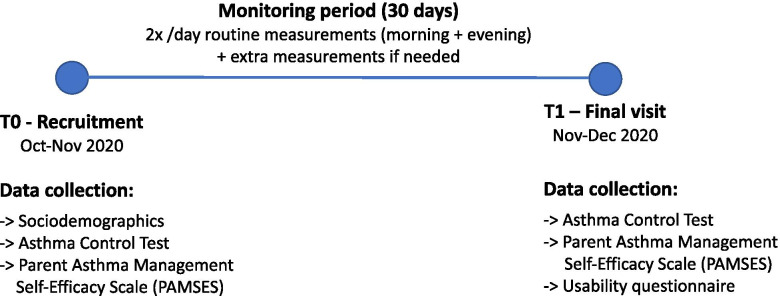
Study timeline. After obtaining written consent from the parents, a recruitment visit (T0) was performed followed by a 30-day monitoring period. The study was finalized for every patient with an individual T1 visit

After obtaining written informed consent, the parents were asked to fill questionnaires on demographics (age, gender, weight and length at birth, siblings) and personal and family anamnesis with regard to atopic diseases, smoke exposure, results from previous allergy diagnostics, clinical characteristics of the child’s wheezing disorder, the asthma control test (ACT) [21], and the Parent Asthma Management Self-Efficacy Scale (PAMSES) [22]. The PAMSES measures parental self-efficacy in preventing and managing their child’s asthma attacks. It consists of 13 items rated on a 5-point Likert scale (1 = not at all sure to 5 = completely sure) with a total possible score range of 13 to 65. After finishing the monitoring period, all families were seen again for a final visit (T1) to assess the ACT, PAMSES, and a usability questionnaire on the use of the device (Fig. 2). The sample size has been determined based on previous experience and according to the recruitment potential of the specialized pediatric center.

### Statistics

Data were summarized as numbers (*n*) and frequencies (%) if categorical and as mean and standard deviation (SD) or median and interquartile range (IQR) if quantitative. Sensitivity and specificity were calculated based on the result of the wheeze detector as the gold standard, as this was previously shown to have a high sensitivity and specificity compared to expert opinion [17]. In this explorative pilot study, descriptive statistics were done using Microsoft® Excel version 16.46.

## Results

### Study population

Twenty children with a mean age of 39.5 months (q1: 24.3 months; q3: 60 months) were included in the pilot study between 1 October and 10 November 2020. Seventeen participants (85%) were male, and 9 (45%) had a family history of atopy (Table 1). The average number of older siblings was 1.2 (SD 1.2), and 5 (25%) children were exposed to tobacco smoke in their homes. Nine of twenty participants had a positive test result either in skin prick testing or serum IgE assessment. During the past 12 months, more than half of the children suffered from dry cough (15, 75%), awakening due to respiratory distress (13, 65%), wheezing after exercise (10, 50%), and a blocked nose (18, 90%). Due to respiratory symptoms, 6 (30%) children had to be taken to the emergency room (ER), 3 (15%) had to be hospitalized, and 10 (50%) missed school or daycare (Table 1). All patients completed the monitoring period, but one family did not attend the final T1 visit.

**Table 1:** Characteristics of the population sample

Male gender (n,%)	17	*85*
Age in months (median, IQR)	39.5	*35.8*
Number of siblings (mean, SD)	1.6	*1.4*
Number of older siblings (mean, SD)	1.2	*1.2*
Smoking at home (n, %)	5	*25*
Family history of atopy (n, %)	9	*45*
Positive SPT/IgE test (n, %)	9	*45*
Use of controller medication	11	*55*
During the past 12 months		
Dry cough (n, %)	15	*75*
Awakening (n, %)	13	*65*
Wheezing after exercise (n, %)	10	*50*
Blocked nose (n, %)	18	*90*
Nebulizer therapy (n, %)	11	*55*
Emergency room visits (n, %)	6	*30*
Hospitalization (n, %)	3	*15*
Missed Day Care (n, %)	10	*50*

### Adherence to symptom and wheeze detector result recording

**Fig. 3:**
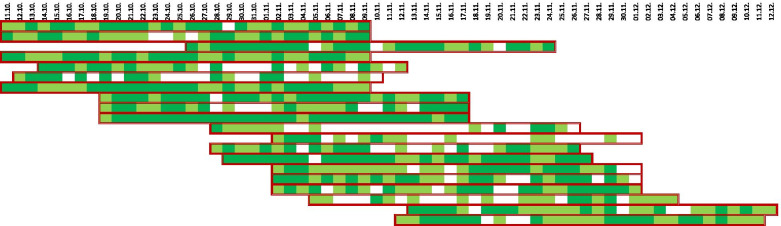
Adherence to compilation of the routine questionnaires (morning and evening) in the WheezeMonitor app. For each patient, a period of 30 days has been considered, starting with the first day of e-diary registration. Days with one registration are marked in light green, while days with complete registration of both questionnaires are marked in dark green. Days with no registration appear in white. The individual monitoring period is marked with a red frame

All participating families were asked to record the child’s respiratory symptoms, as well as the WheezeScan measurement results and whether they administered reliever medication at least twice a day. Although adherence to recording varied (Fig. 3), the participants filled at least one questionnaire in 24/30 (81%) of the days (Table 2). The average number of days with complete data recording was 13.8, so adherence to complete monitoring was 45.8% on average. The recording of two data sets per day was more frequent than unique data entry, which was performed on an average of 10.6 out of 30 days (35.2%). Several mothers reported delayed symptom recording in the app due to their busy family routine. This was also mirrored in the timestamps of the recorded data sets.

**Table 2:** Number (%) of days with one, two, or at least one completed questionnaire

**Patient No.**	Diary filled 1x/d		Diary filled 2x/d		Diary filled at least 1x/d	
	n	%	n	%	n	%
**1**	13	*43.3*	16	*53.3*	29	*97*
**2**	15	*50.0*	12	*40.0*	27	*90*
**3**	7	*23.3*	20	*66.7*	27	*90*
**4**	14	*46.7*	16	*53.3*	30	*100*
**5**	10	*33.3*	11	*36.7*	21	*70*
**6**	5	*16.7*	10	*33.3*	15	*50*
**7**	12	*40.0*	18	*60.0*	30	*100*
**8**	9	*30.0*	21	*70.0*	30	*100*
**9**	11	*36.7*	13	*43.3*	24	*80*
**10**	3	*10.0*	27	*90.0*	30	*100*
**11**	8	*26.7*	4	*13.3*	12	*40*
**12**	9	*30.0*	4	*13.3*	13	*43*
**13**	11	*36.7*	11	*36.7*	22	*73*
**14**	6	*20.0*	23	*76.7*	29	*97*
**15**	15	*50.0*	11	*36.7*	26	*87*
**16**	10	*33.3*	15	*50.0*	25	*83*
**17**	11	*36.7*	14	*46.7*	25	*83*
**18**	14	*46.7*	4	*13.3*	18	*60*
**19**	14	*46.7*	12	*40.0*	26	*87*
**20**	14	*46.7*	13	*43.3*	27	*90*
**Average**	10.6	*35.2*	13.8	*45.8*	24.3	*81.0*
**SD**	3.5	*11.6*	6.1	*20.4*	5.7	*19.1*

### Parental and doctor’s clinical evaluation versus wheeze detector results

In order to avoid any influence of the results given by the wheeze detector on the parents’ perception of their child’s clinical condition, we clearly instructed all participants on first recording their subjective impression on the presence or absence of respiratory symptoms before using the wheeze detector. In a total of 708 recordings, parents pre-detected wheezing in 22 cases (3.1%) while the wheeze detector did so in 140 measurements (19.8%). This resulted in a sensitivity of parental judgment of 15%, while specificity was 99.8%. To compare the study doctor’s (a specialized pediatric pulmonologist) assessment of current wheezing with the wheeze detector results, we asked the study team to measure and record both results whenever a participant came to the office for an unscheduled visit (independently of the reason for the visit). Over the entire study period, 13 unscheduled visits were recorded with a sensitivity of the doctor’s assessment of 83.3% and a specificity of 100%. The positive (PPV) and negative predictive values (NPV) for parents were 95.5% and 82.7%, respectively. The comparison of the wheeze detector results with the doctor’s judgment resulted in a PPV of 83.3% and an NPV of 87.3%.

### Impact of a digital wheeze detector on parental self-efficacy and asthma control

**Fig. 4:**
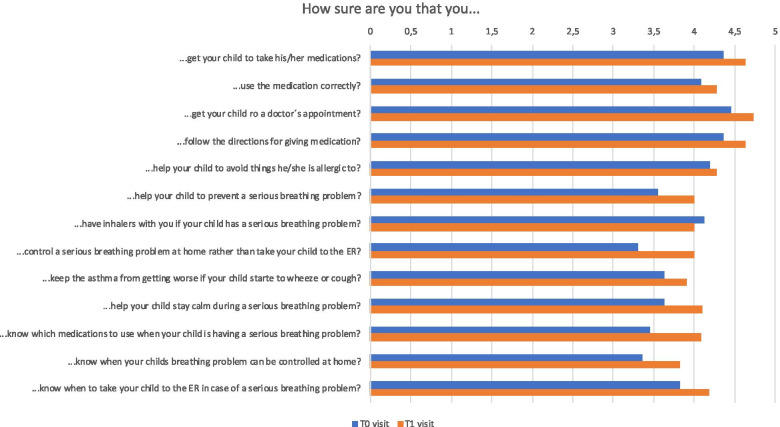
Results of the Parent Asthma Management Self-Efficacy Scale (PAMSES) measuring parent self-efficacy in preventing and managing children’s asthma attacks. The PAMSES consists of 13 items rated on a 5-point Likert scale (1 = not at all sure to 5 = completely sure) with a total possible score range of 13 to 65

The trend in asthma control test could be evaluated for 14 patients after removing those with an incomplete data set. Over the observation period of 30 days, the asthma control test improved for 9/14 (64%) patients from 18.1 to 22.6 points on average. One patient remained stable, and 4 patients decreased their score from 18 to 16.25 points on average. Regarding the results of the PAMSES questionnaire, improvements could be observed for all items apart from one (“How sure are you that you have inhalers with you if your child has a serious breathing problem?”). The most prominent improvement (0.7 points) concerned parental security in treating their child’s serious breathing problem at home rather than taking the child to the ER (Fig. 4).

### Usability and perceived benefits/risks of the digital wheeze detector

**Fig. 5:**
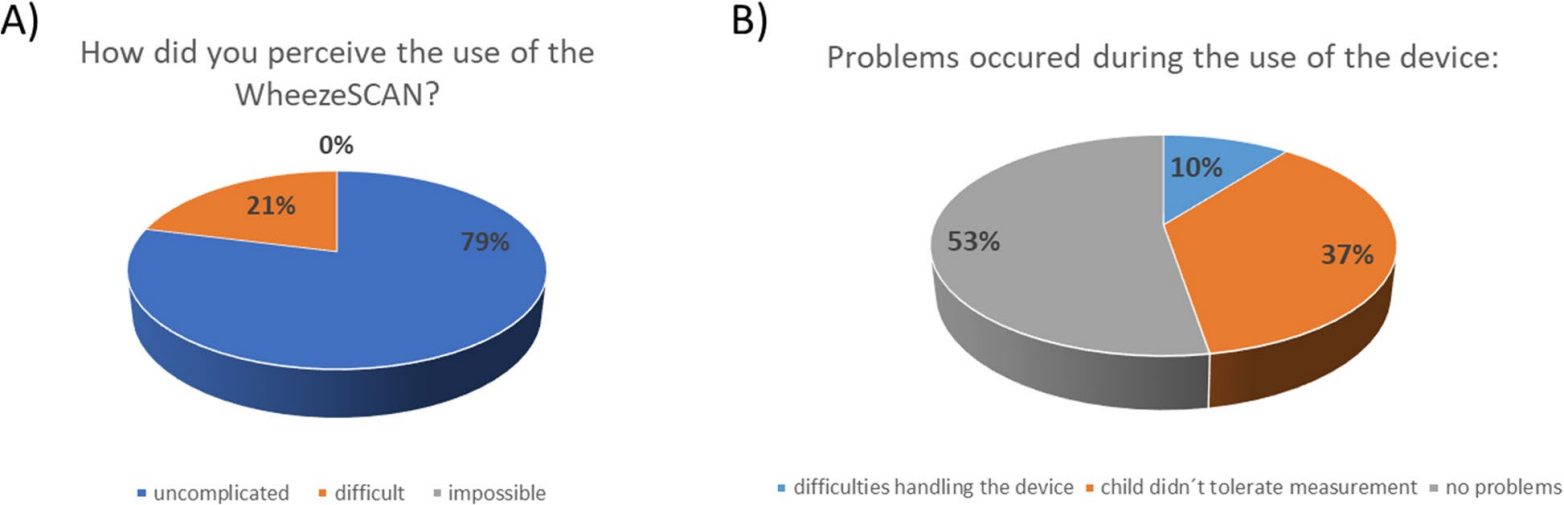
Parental usability evaluation of the digital wheeze detector

**Fig. 6:**
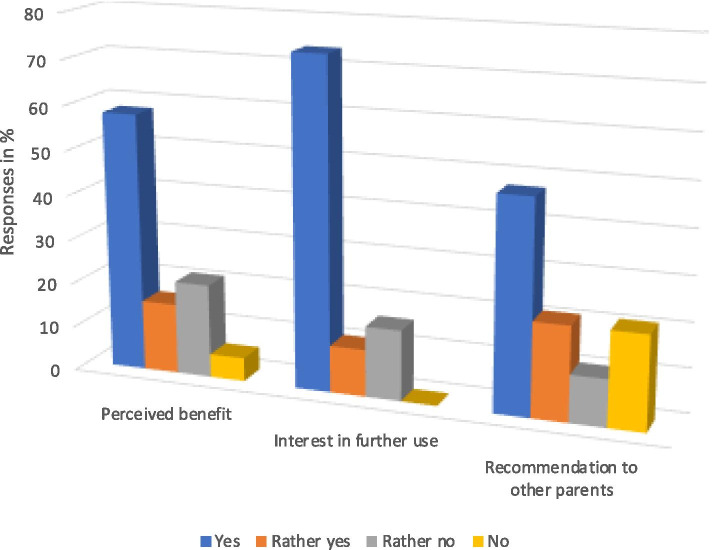
Results of the parents’ perceived benefit and recommendability of the digital wheeze detector. Parents were asked whether they (i) perceived a benefit of the use for their own child, (ii) would like to keep using the device in the future, and (iii) would recommend the device to other parents

Overall, 15/19 (79%) of the participants described the use of the wheeze detector as uncomplicated, while only 4/19 (21%) described the handling as “difficult” (Fig. 5a). Two of 19 (11%) parents reported difficulties in the use of the device, and 7/19 (37%) had “troubles in keeping their child calm enough.” In many cases, the child could not sit still for 30 s, which is needed for the device to assess breathing sounds (Fig. 5b). Several (10/19, 53%) parents perceived the device as overly sensitive to ambient sounds, which led to an indication of an error on the instrument display and the need for repeated measurements. Last, the device took longer to generate an outcome, if wheezing was absent or mild, while clinically more prominent wheezing was detected within a few seconds only. Most families were happy using the device and perceived it as beneficial for their children. Interestingly, more than 70% of the parents wanted to keep the wheeze detector for use in the future, and 45% would recommend it to other parents of wheezing children (Fig. 6).

## Discussion

In our exploratory pilot study on the impact of a digital wheeze detector on the home management of wheezing disorders in pre-school children, we observed that (i) the recruitment procedures were feasible, (ii) the use of the WheezeScan detector is easy and safe for children with wheezing aged 9 to 70 months, (iii) parents reliably record symptoms and wheeze detector results over a time span of 30 days via eDiary, and (iv) the support of a digital wheeze detector improves parental self-efficacy in asthma/wheeze management.

Concerning the demographic and clinical characteristics of our pilot cohort, we observed a predominance of the male gender, which reflects epidemiological data on asthma prevalence observed in several waves of a large population-based cohort study in Germany [23]. Common risk factors for the development of wheezing and/or asthma, such as exposure to tobacco smoke, family history of atopy, and positive skin prick or IgE testing were observed in 45% of the participants, meeting the expectations based on current knowledge [24, 25].

The recruitment and inclusion procedures in a private outpatient clinic were feasible. Interestingly, parental identification of wheezing was less frequent than expected during winter months: only 22/708 (3.1%) single events were recorded by the parents, increasing to 140/708 (19.8%) observations of wheezing when using the digital wheeze detector. This confirms previous observations of potential parental insecurity in the clinical evaluation of their child [26] and underlines the need for a larger study to assess the benefit of digital support in a home care setting of pre-school wheezing. In addition, the subjective impression of parents participating in this pilot study reflected that many participants felt more secure in the management of their child’s wheezing disorder at home when using a digital support tool. This is also shown by the improvement of the PAMSES Score between the T0 and T1 study visits (Fig. 4). When comparing the study doctor’s detection of wheezing with the WheezeScan, sensitivity increased to 83.3% with a specificity of 100%. These data are in accordance with previously described concordances between the doctor’s evaluation and the results of the device [17]. However, it needs to be considered, that this comparison is based on a low number of unscheduled visits (*n* = 13), which is most likely due to mitigation measures taken in the current SARS-CoV-2 pandemic.

An important aim of this pilot study was to observe the adherence to the use of the wheeze detector and subsequent recording via eDiary. Especially the measurement with the WheezeSCAN® may affect the families’ daily life as the child needs to remain calm and surroundings as silent as possible for approximately 30 s to allow measuring. While the adherence to measuring and complete recording (2×/day) reached an average of 45.8%, parents recorded at least once daily on an average of 24.3/30 (81%) of the days. This corresponds to previously observed adherence data for symptom monitoring via eDiary [27]. As some families reported delayed data entry in the study app, a certain recollection bias may be possible. Concerning the usability of the device, most 79% of the families rated the technical use as “uncomplicated” (Fig. 5a). Those indicating difficulties specified mostly (37%) problems in convincing their child to remain calm and tolerate the measurement (Fig. 5b). Interestingly, the reporting of this difficulty did not relate to the age of the children or the number of siblings as a potential cause for interfering noises. Despite the described individual challenges, participants rated the overall usability as high and no serious technical problems occurred.

In summary, the usability of WheezeSCAN and the adherence to its use and to an eDiary recording were high, but a longer observation period, ideally without broad contact restrictions and pandemic mitigation measures, will be necessary in order to observe more wheezing episodes. This pilot experience suggests that WheezeSCAN® may empower parents by increasing their capacity for wheeze detection. The recruitment and monitoring procedures were safe and feasible; hence, the usability and impact of the wheeze detector deserve to be further investigated in a large, multicenter, randomized, controlled trial.

## Data Availability

The collected data and material are safely stored on Charité servers in order to protect the participants’ rights in compliance with the GDPR.

## Abbreviations

ACT: Asthma control test
eDiary: Electronic diary
ER: Emergency room
IgE: Immunoglobulin E
IQR: Interquartile range
LRI: Lower respiratory tract infection
NPV: Negative predictive value
PAMSES: Parent Asthma Management Self-Efficacy Scale
PPV: Positive predictive value
RCT: Randomized controlled trial
SD: Standard deviation
SPT: Skin prick test
URI: Upper respiratory tract infection
